# Innovative *in vivo* rat model for global cerebral hypoxia: a new approach to investigate therapeutic and preventive drugs

**DOI:** 10.3389/fphys.2024.1293247

**Published:** 2024-02-09

**Authors:** Sarah Stahlke, Jonas Frai, Johanna Franziska Busse, Veronika Matschke, Carsten Theiss, Thomas Weber, Jennifer Herzog-Niescery

**Affiliations:** ^1^ Institute of Anatomy, Department of Cytology, Ruhr-University Bochum, Bochum, Germany; ^2^ Department of Anesthesiology and Intensive Care Medicine, St.Josef-Hospital Bochum, Bochum, Germany

**Keywords:** cerebellum, global cerebral hypoxia, hippocampus, purkinje cells, rat animal model, rocuronium, sugammadex

## Abstract

**Introduction:** Severe acute global cerebral hypoxia can lead to significant disability in humans. Although different animal models have been described to study hypoxia, there is no endogenous model that considers hypoxia and its effect on the brain as an independent factor. Thus, we developed a minimally invasive rat model, which is based on the non-depolarizing muscle blocking agent rocuronium in anesthetized animals. This drug causes respiratory insufficiency by paralysis of the striated muscles.

**Methods:** In this study, 14 rats underwent 12 min of hypoxemia with an oxygen saturation of approximately 60% measured by pulse oximetry; thereafter, animals obtained sugammadex to antagonize rocuronium immediately.

**Results:** Compared to controls (14 rats, anesthesia only), hypoxic animals demonstrated significant morphological alterations in the hippocampus (cell decrease in the CA 1 region) and the cerebellum (Purkinje cell decrease), as well as significant changes in hypoxia markers in blood (Hif2α, Il1β, Tgf1β, Tnfα, S100b, cspg2, neuron-specific enolase), hippocampus (Il1β, Tnfα, S100b, cspg2, NSE), and cerebellum (Hif1α, Tnfα, S100b, cspg2, NSE). Effects were more pronounced in females than in males.

**Discussion:** Consequently, this model is suitable to induce hypoxemia with consecutive global cerebral hypoxia. As significant morphological and biochemical changes were proven, it can be used to investigate therapeutic and preventive drugs for global cerebral hypoxia.

## 1 Introduction

Acute severe global cerebral hypoxia, e.g., following peripartum asphyxia, carbon monoxide intoxication, or respiratory insufficiency of other origin, impairs the cerebral cell metabolism with increased anaerobic glycolysis and lactate production, affects the transmitter activity as well as extra- and intracerebral homeostasis, and causes significant morbidity and mortality in humans (Canazza et al., 2014).

Different animal models for cerebral hypoxia have been developed in recent years to study the underlying disease-specific neuronal pathophysiology, adaptive processes after cerebral hypoxia, and for assessing the potential of novel therapeutic strategies. Although in some hypoxia models larger mammals like sheep and monkeys were used (Clapp et al., 1988; Güzel et al., 2014), rats are often seen as species of choice, because their neurovascular branching is comparable to humans and reproducibility of experimental results is high (Canazza et al., 2014). Moreover, global cerebral hypoxia models in large animals are challenging regarding equipment, costs, personnel, and surgical experience (Gunn et al., 2000).

In literature available hypoxia rat models were recently systematized in a review article (Salyha and Oliynyk, 2023). The authors present a differentiation in exogenous hypoxia models, including hypobaric (decreased oxygen partial pressure accompanied by a changed barometric air pressure) or normobaric (percentage drop in oxygen in the inhaled air at a barometric pressure of 750 mmHg caused by an altered gas composition) methods, and endogenous hypoxia models, which mimic the pathophysiology of the investigated diseases. All these models have inherent advantages and disadvantages, however, there is no endogenous model that considers hypoxia and its effect on the brain as an independent factor. There are of course models that focus primarily on ischemic brain injury, e.g., caused by temporarily or permanently surgical occlusion of intracerebral blood vessels, but the resulting ischemia and reperfusion is not the pathophysiological mechanism of global cerebral hypoxia with still preserved cerebral blood flow (Brambrink et al., 1999). Consequently, these models are neither suitable nor intended to investigate cerebral hypoxia. Additionally, most existing endogenous models do not develop hypoxia rapidly, but only after hours (Koehler et al., 2018), the hypoxic effect is difficult to control or need secondary stimuli (Brann and Myers, 1975), some models require surgery with relevant side-effects, are labor-intensive (Kim et al., 2016), or the rats die within minutes due to the procedure (e.g., induction of methemoglobinemia), which makes it impossible to address the long-term outcome after a pharmacological treatment (Tran et al., 2015).

In the clinical setting patients sometimes suffer a very acute hypoxic event for a short period of time, which often goes along with dramatic neurological consequences. Beside the above mentioned examples, this also includes bolus aspiration or so called “cannot ventilate, cannot intubate-situations” during induction of anesthesia, in which the patient can no longer breathe independently, but the airway cannot be secured either. Thus, we were interested in an endogenous rat model for global cerebral hypoxia, which mimics a rapidly developing hypoxic event with clearly defined beginning and end due to respiratory insufficiency, in which the animals recover clinically to such an extent that a follow-up monitoring over a longer period of time is possible.

Therefore, we developed a minimally invasive rat model, which is based on the working principle of the non-depolarizing muscle blocking agent rocuronium. This drug is used in anesthesia for about 30 years to optimize working conditions for anesthesiologists and surgeons by paralyzing the striated muscles (Hawkins et al., 2019). However, rocuronium also relaxes the respiratory muscles (diaphragm and other auxiliary respiratory muscles), which leads to insufficient ventilation up to a complete respiratory arrest about 2–3 min after application (Hausmann et al., 2007). If an organism is then not mechanically ventilated, a lack of oxygen develops, resulting in hypoxemia and global cerebral hypoxia. An easy way to antagonize rocuronium is the administration of the relaxant binding agent sugammadex, which encapsulates rocuronium within seconds and immediately cancels its effects. The organism starts breathing again (Sekhon et al., 2017). The binding between rocuronium and sugammadex is very strong and cannot be solved easily, neither spontaneously nor by any of the drugs administered in this experiment (dos Santos et al., 2022). The complex of rocuronium and sugammadex is then eliminated biliary and by renal excretion. A residual neuromuscular, which may occur shortly after antagonization, results from an insufficient sugammadex dosage and can easily be avoided by continuous monitoring of the animals until they are awake. Other “long-term side-effects” of rocuronium are not to be expected.

This principle allows us to focus on the effect of global cerebral hypoxia only, as the cerebral blood flow is maintained. This manuscript explains the technical details of the model and demonstrates morphological and biochemical results after 12 min of hypoxemia with an oxygen saturation of about 60% measured by pulse oximetry.

## 2 Material and equipment

### 2.1 Animals

All procedures were carried out under established standards of the German federal state of North Rhine Westphalia, in accordance with the European Communities Council Directive 2010/63/EU on the protection and care of animals used for scientific purposes. The permission to conduct these animal experiments was granted by the North Rhine-Westphalia State Office for Nature, Environment and Consumer Protection (LANUV-NRW), file no. 81-02.04.2021. A452.

The Wistar rats used in this study were bred in-house at the animal facility of medicine of the Ruhr-University Bochum, following the selection of breeding pairs based on age, weight, and overall health. Adhering to the principles of the 3Rs, the breeding program aimed to minimize unnecessary animal use by breeding only the required number of animals for the study, resulting in a lack of influence over sex distribution. For female rats it was ensured that they were not pregnant before or during the experiments. Animal care procedures followed ethical guidelines and regulations. Rats were kept under a 12-h light/dark cycle and *ad libitum* access to food and water. A total of 28 rats, aged 16 weeks, were used, with sexes evenly distributed among the experimental groups.

### 2.2 Reagents and equipment

Reagents and equipment necessary for induction of global cerebral hypoxia is listed in Table 1 in the order of use as indicated in the Methods section. Note that the method of anaesthesia and corresponding reversion can be adapted according to the needs of the experiment. Here, the shortest possible anaesthesia was chosen to ensure the lowest possible stress for the animals according to the 3R principle.

**TABLE 1:** Used regents and equiment for induction of global cerebral hypoxia and following experiments for validation.

Reagent/Equipment	Supplier	Working dilution
Essential materials used for global cerebral hypoxia		
ketamine	Ketabel^®^, Bela-Pharm GmbH, Germany	50 mg/kg
midazolam	Dormicum^®^, ratiopharm GmbH, Germany	2.5 mg/kg
pulse oximeter	VET Veterinary Oximeter, UNO Live Science Solutions, Netherlands	---
Heat-mat	UNO Live Science Solutions, Netherlands	---
O_2_ mask	UNO Live Science Solutions, Netherlands	---
rocuronium	Esmeron^®^, hameln-pharma, Germany	40 mg/kg
sugammadex	Bridion^®^, MSD, Austria	66 mg/kg
flumazenil	Anexate^®^, HIKMA Pharma GmbH, Germany	0.25 mg/kg
doxapram	Dopram^®^, Dechra, Germany	1 drop
Additional materials used for validation/follow up experiments		
monovettes	S-Monovette EDTA 1.6 mL, Sarstedt, Nümbrecht, Germany, 05.1081.001	---
NucleoSpin Blood Kit	740,951.10, Macherey-Nagel, Düren, Germany	---
reverse transcriptase	GoScriptTM Reverse Transcription Mix, Oligo (dT), A2790 - Promega, Madison, WI, United States	---
GoTaq^®^ qPCR Master Mix	A6001, Promega, Madison, WI, United States	---
CFX Connect Real Time PCR Detection System	Bio-Rad, Hercules, CA, United States	---
PFA	Paraformaldehyde,28,794.295, VWR Chemicals, Darmstadt, Germany	4% (in PBS)
sucrose	D (+)-Sucrose pure, A1125, AppliChem, Darmstadt, Germany	20% (in PBS)
cryostat	CryoStar NX50, Thermo Scientific, Germany	---
freezing medium	No. 14020108926, Leica Biosystems	---
Superfrost-Plus Adhesion Slides	J1800AMNZ, Thermo Scientific, Germany	---
cresyl violet	7651.1, Carl Roth, Karlsruhe, Germany	0.25% cresyl violet in 100% ethanol (EtOH) and distilled water
Cover slips	Menzel 24 × 50 mm, Thermo scientific, Germany	---
mounting medium	Eukitt^®^ quick-hardening mounting medium, Sigma-Aldrich, Germany	---
fluorescence microscope	BZ-X800, Keyence, Germany	---

## 3 Methods

### 3.1 Experimental setup - induction of global cerebral hypoxia

**FIGURE 1:**
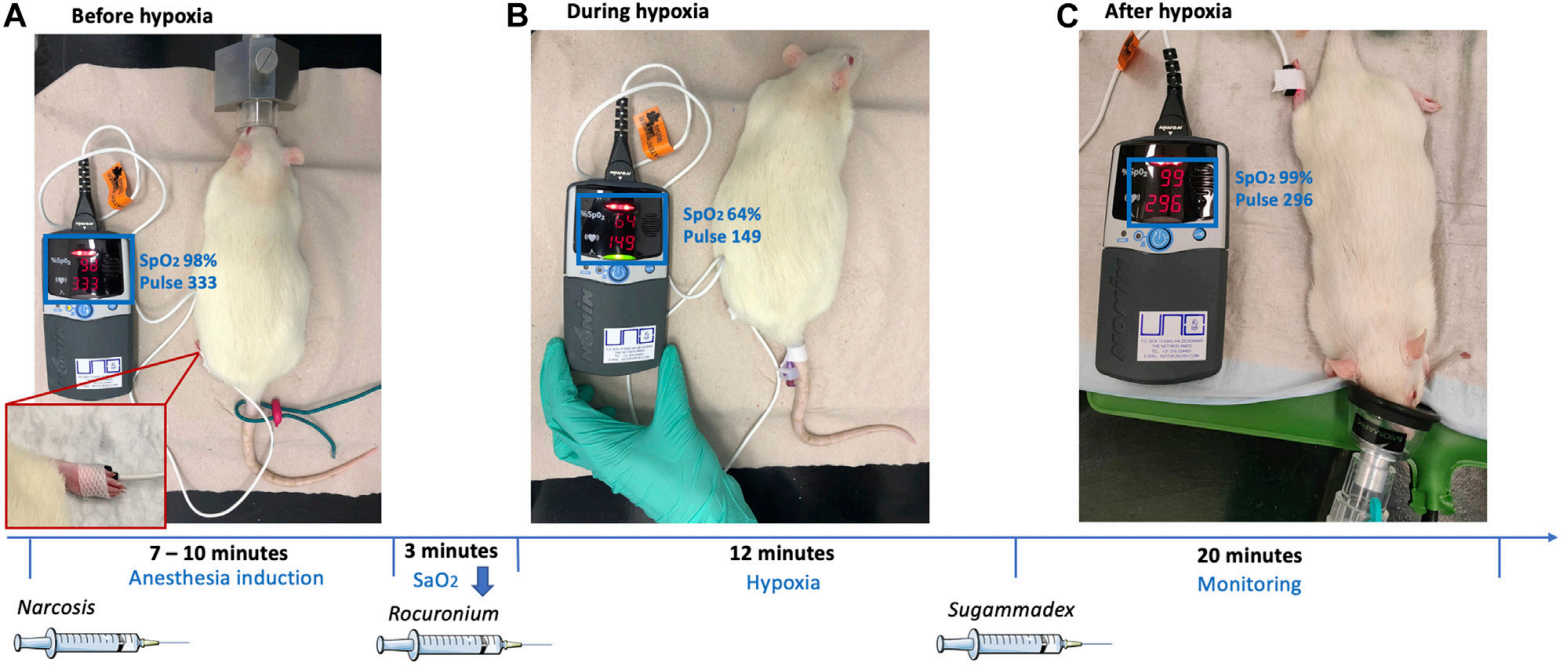
Exemplary representation of the experimental setup, showing the rat before, during and after hypoxia and additional timeline.

Rats were anesthetized by i. p. application of 50 mg/kg ketamine and 2.5 mg/kg midazolam. A pulse oximeter was fixed on a hind paw to monitor the oxygen saturation and the heart rate continuously (Figure 1, lower left corner). The body temperature was measured and regulated using a heat-mat (green, Figure 1, right). Once the oxygen saturation dropped below 91% animals obtained oxygen through a small animal mask until an oxygen saturation above 90% was maintained. After the absence of reflexes was verified, an intravenous catheter was placed (via vena caudalis lateralis; Figure 1, middle) and 40 mg/kg rocuronium was injected intravenously. The oxygen supply was stopped, and the saturation dropped continuously because of the reduced tidal volume. An oxygen saturation of 60% was aimed. Once a 10% reduction compared to the baseline saturation was reached (oxygen saturation always <90%), the “hypoxemia interval” started. This condition was maintained for 12 min, before 66 mg/kg sugammadex i. v. was administered to encapsulate rocuronium and to antagonize its effect. Animals further obtained 0.25 mg/kg flumazenil i. v. to antagonize midazolam, a drop of oral doxapram for central respiratory stimulation, and oxygen to ensure an oxygen saturation ≥91%. Animals were monitored until they were fully awake.

The control group underwent the same anesthesiological procedure but obtained neither rocuronium nor sugammadex. The oxygen saturation remained above 90% throughout the procedure.

All animals were terminated 24 h after induction of anesthesia. Blood and brain tissues were collected for further immunohistochemical and molecular analysis.

Note: The length of relaxation by rocuronium can be adjusted according to the research question. Initially, 12 min were chosen, as this corresponds to approximately the maximum time a human can survive without oxygen but will suffer damage to the brain tissue.

### 3.2 Validation

#### 3.2.1 RNA and cDNA extraction

Blood was taken directly from the heart and collected in 1.6 mL monovettes. cDNA was purified from each 200 µL blood to focus on gene expression at the transcript level by using a NucleoSpin Blood Kit according to the manufacturer’s instructions.

The hippocampus and the cerebellum were dissected from one hemisphere of each brain. The right hemisphere of every animal was used for RNA isolation, of which the whole hippocampus and cerebellum were dissected on ice. When dissecting and isolating the hippocampus from the rest of the brain tissue, it is crucial to carefully identify the anatomical landmarks that define the boundaries of the hippocampus. These landmarks may vary slightly depending on the dissection technique. In general, the following landmarks may be used for identification of the hippocampus: 1. The external appearance, as the hippocampus generally exhibits a curved “C”-shape; 2. The hippocampus is located in the medial temporal lobe of the brain; 3. The fornix is a bundle of nerve fibers that runs along the ventral surface of the hippocampus; 4. The dentate gyrus is a distinct structure within the hippocampus. It appears as a curved band of cells on the dorsal surface of the hippocampus. Both structures were homogenized completely but only the amount of tissue suggested by the manufacturer employed for RNA extraction. The RNA of each area was isolated separately using the NucleoSpin RNA Kit (740,955.50, Macherey-Nagel, Düren, Germany). In deviation from the manufacturer’s instructions, RNA was eluted in only 30 µL of nuclease-free water as a last step. 1 μg of the resulting RNA was transcribed into cDNA using reverse transcriptase according to the manufacturer’s instructions.

#### 3.2.2 Quantitative real-time PCR (qRT-PCR)

GoTaq^®^ qPCR Master Mix was used to perform qRT-PCR according to the manufacturer’s instructions. Blood-derived cDNA was diluted 1:15 and tissue-derived cDNA was diluted 1:20 in nuclease-free water. Expression levels for the genes of interest and for housekeeping gene GAPDH were measured in at least duplicates. Used primers are demonstrated in Table 2. Quantitative RT-PCRs were performed on a CFX Connect Real Time PCR Detection System. The X0 method [“Analysis of qPCR data by converting exponentially related Ct values into linearly related X0 values” (Thomsen et al., 2010);] was applied, using the mean ct-value normalized to the housekeeping gene GAPDH, before the results were plotted logarithmically to base 10 using mean and standard deviation (SD). Actin was used as an additional housekeeping gene in early experiments (data not shown), but turned out not to be stably expressed, so only GAPDH was used.

**TABLE 2:** List of primers used to validate successful global cerebral hypoxia.

Primer	Forward	Reverse
Hif1α	AAT​GTA​CCC​TAA​CAA​GCC​GGG	GTT​TCT​TGT​AGC​CAC​ACT​GCG
Hif2α	AGT​GGT​CTG​TGG​GCA​ATC​AG	AAC​ATG​GAG​ACA​TGA​GGC​GG
Il1β	GAC​AAG​AGC​TTC​AGG​AAG​GCA	CCA​CGG​GCA​AGA​CAT​AGG​TAG
Tgfβ1	CTG​CTG​ACC​CCC​ACT​GAT​AC	AGC​CCT​GTA​TTC​CGT​CTC​CT
Tnfα	GCT​CCC​TCT​CAT​CAG​TTC​CA	GCTACGGGCTTGTCACTC
S100b	CTG​GAG​AAG​GCC​ATG​GTT​GC	CTC​CAG​GAA​GTG​AGA​GAG​CT
cspg2	CGC​CTA​AGA​CAC​TAC​GTA​TGC​TTG​T	TTG​GTC​CTA​TGT​TGA​CTG​TTT​CTC​A
GAPDH	GGG​TGT​GAA​CCA​CGA​GAA​AT	ACT​GTG​GTC​ATG​AGC​CCT​TC
NSE	GGG​GCA​CTC​TAC​CAG​GAC​TTT​G	GTT​CCG​GTG​TTC​AGG​CAA​GCA​G

#### 3.2.3 Cryosectioning

The left hemisphere of every animal was fixed in 4% PFA immediately after preparation. Once it was saturated (approximately after 24–48 h), the tissue was transferred to 20% sucrose and stored at 4°C.

The complete hemispheres were cut into 18 µm sagittal sections, with 3 sections per slide grouped in slides of 10. For RNase-free conditions removable parts of the cryostat were cleaned with NaOH-EDTA. Cryosectioning was performed at a chamber temperature of −20°C and a stage temperature of −18°C to −16°C. In the chamber the frozen hemispheres were stabilized on the microscope slide with tissue freezing medium. After 20–30 min acclimation time it was attached to the object holder of the cryostat with the lateral part facing up and cut. To ensure uniformity between the rats, serial brain slices were created, each labeled with chronological numbers. In total, 40 slides, each containing three brain slices from the individual rat were created (see above). In total 120 brain slices per rat were taken, giving an overview over the whole hemisphere. For the research model the brain slices corresponding to slide numbers 10, 20, 30, and 40 were analyzed.

The serial cryosections for immunohistochemistry were mounted on Superfrost-Plus Adhesion Slides and stored at 4°C until further use.

#### 3.2.4 Cresyl violet staining

For histochemical staining the cryosections were submerged in cresyl violet-stain for 2 minutes. The slides were then rinsed with tap water to remove any excess stain in two different cuvettes. To decolorize the slides, they were washed two times with 95% EtOH for 10–15 s. To differentiate them further, the samples were twice re-washed with 100% EtOH for 2.5 min. Then, the slides were placed in a cuvette with 100% xylol for 2.5 min. After differentiation the stained samples were cover slipped with mounting medium.

#### 3.2.5 Determining the cell numbers in the hippocampus and the cerebellum

The CA1 region in cresyl violet-stained slides was analyzed using the same exposure time for every slide with the fluorescence microscope and an objective lens magnification of ×20. The obtained pictures were imported to ImageJ Software (Schneider, C.A., Rasband, W.S., Eliceiri, K.W. “NIH Image to ImageJ: 25 years of image analysis”. Nature Methods 9, 671-675, 2012) and converted to greyscale (16-bit). The threshold was adjusted, so that it highlighted all cell nuclei in the CA1 region. A square was drawn around the CA1 region and the mean gray value (representing cell numbers) within this area was calculated (compare Figure 3). The evaluated surface area of the hippocampus mean gray value measured 276 mm^2^.

Analogous to the procedure described above, the images for the evaluation of Purkinje cell numbers in ImageJ were captured. Again, pictures were converted to greyscale. For cell counting, the multi-point tool was used, capturing every Purkinje cell along the primary fissure.

To ensure a non-biased evaluation, a single observer conducted both observations, intentionally blinded to the experimental conditions.

For further classification the Purkinje cells were divided into three categories according to their cytological characteristics: intact Purkinje cells with distinct stained nuclei (PC Type I), necrotic/swollen Purkinje cells with a lack of nuclear staining and distinct cellular morphology (PC Type II) and shrunken disfigured cells with intensive (dark) staining of the cytoplasm (PC Type III). Image scanning was performed with ZEISS ZEN software; a unit length of 1,000 µm was used. Again, the Purkinje cells were counted along the primary fissure of the cerebellum (assisted by ImageJ multi-point tool and marked by mouse click) and allocated to PC groups I-III. Furthermore the area of the Purkinje cell body was outlined with the freehand tool in ImageJ. The measure feature was used to analyze the area, shape descriptors, perimeter, and Feret’s diameter of the cells.

#### 3.2.6 Statistical analyses

Data obtained from the study were analyzed using appropriate statistical methods, such as t-tests for intergroup differences or analysis of variance (ANOVA), when examined more than two groups. Before the statistical analyse were conducted we analysed whether the data had a Gaussian distribution, which was the case. Results were reported as mean ± standard deviation, and significance levels were set at *p* < 0.05 indicated with *, *p* < 0.001 indicated with ** or *p* < 0.0001 indicated with ***. The data obtained in this study were subjected to additional analysis to identify potential statistical outliers. The ROUT method, with a Q-value set at 5%, was employed for this purpose. This analysis allowed the detection and potential exclusion of data points that significantly deviated from the majority of the dataset, ensuring the robustness and reliability of the statistical analysis.

The results were plotted as mean ± standard deviation (SD), using GraphPad Prism9 (GraphPad Software, San Diego, California United States, www.graphpad.com).

## 4 Results

### 4.1 Hypoxemia in investigated animals

Fourteen rats were investigated; 7 of them were male and 7 female. After induction of anesthesia, the mean oxygen saturation level recorded by pulse oximetry (SaO_2_) was 98% (±2.7). Once rocuronium was administered, a SaO_2_ reduction by 10% was reached after 3.1 min (±1.7), which was the starting point of the 12 min hypoxemia interval. The SaO_2_ dropped continuously to an average of 62% (±5.8) and stabilized without any differences between the sexes (*p* = 0.676). There was also no correlation between the markers examined and the percentage decrease in the measured oxygen saturation (see supplementary).

After 12 min of hypoxemia, the muscle-relaxing effect of rocuronium was antagonized by sugammadex. It took 1.6 min (±0.6) on average to reach the SaO_2_ baseline. Again, no significant differences between the sexes were observed (compare Table 3 for details).

**TABLE 3:** Details of investigated animals suffering 12 min of hypoxemia.

No.	Sex	Measured SaO2 max [%]	10% decrease SaO_2_ [min]	Measured SaO_2_ minimum [%]	Total decrease SaO_2_ referring to baseline [%]	Recovery time [min]	Measured SaO_2_ end [%]
1	m	100	2	61	39	1	93
2	m	100	1	61	39	3	95
3	m	94	5	66	30	2	91
4	m	96	3	62	35	1	99
5	m	100	3	72	28	1	99
6	m	98	2	72	27	1	99
7	m	97	1	50	48	2	100
8	f	100	2	59	41	2	100
9	f	100	7	61	39	2	96
10	f	100	2	61	39	1	100
11	f	98	4	66	33	2	96
12	f	91	6	65	29	2	96
13	f	100	3	58	42	1	97
14	f	99	3	68	32	1	98
Control group: 14 additional animals, 7 female and 7 male							

The control group consisted of 14 rats, 7 of them female and 7 male. These animals obtained anesthesia only. The SaO2 was always above 90%.

### 4.2 Morphological areas of interest

**FIGURE 2:**
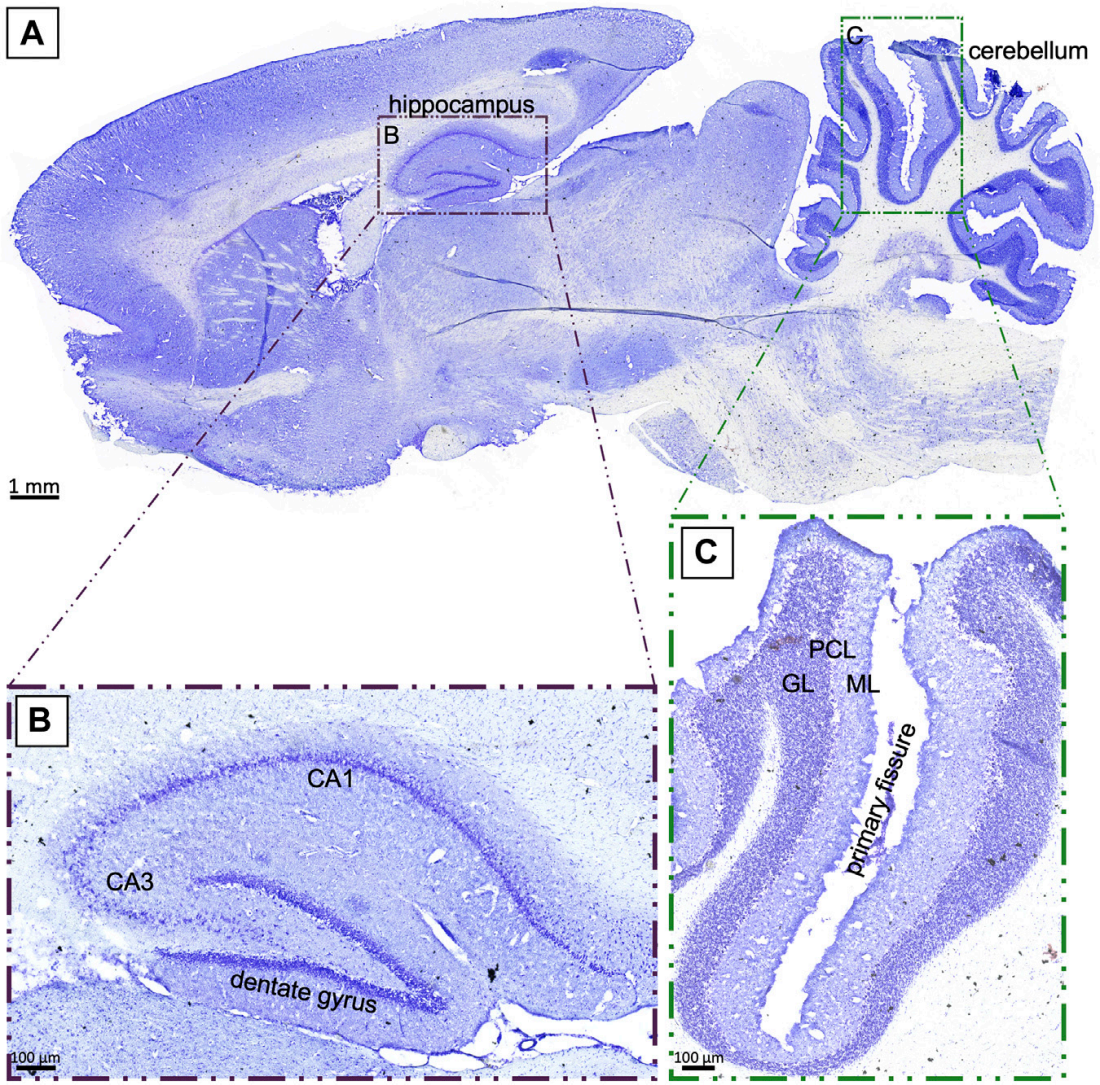
**(A)**: Sagittal section of a control rat brain in cresyl violet-staining. The investigated regions of the hippocampus **(B)** and the cerebellum **(C)** are highlighted. GL: granule layer; PCL: Purkinje cell layer; ML: molecular layer; scale bar in **(A)** 1 mm; **(B)**, **(C)** 100 µm.

Global cerebral hypoxia affects the entire brain, but regions such as the hippocampus and the cerebellum are known to be particularly vulnerable to a lack of oxygen (Schmidt-Kastner and Freund, 1991; Brasko et al., 1995; Barenberg et al., 2001; Bartsch and Wulff, 2015). Figure 2A provides a sagittal overview of a control rat brain in cresyl violet-staining, including 40-fold magnifications of the CA1 region (hippocampus, Figure 2B) and the primary fissure (cerebellum, Figure 2C) for orientation. More detailed analysis demonstrated densely packed nuclei in the CA1 region of the hippocampus, as well as a clear subdivision into granule, purine, and molecular cell layers along the primary fissure of the cerebellum, which both are typical observations in an intact rat brain.

#### 4.2.1 Morphological alterations in the CA1 region of the hippocampus after hypoxemia

**FIGURE 3:**
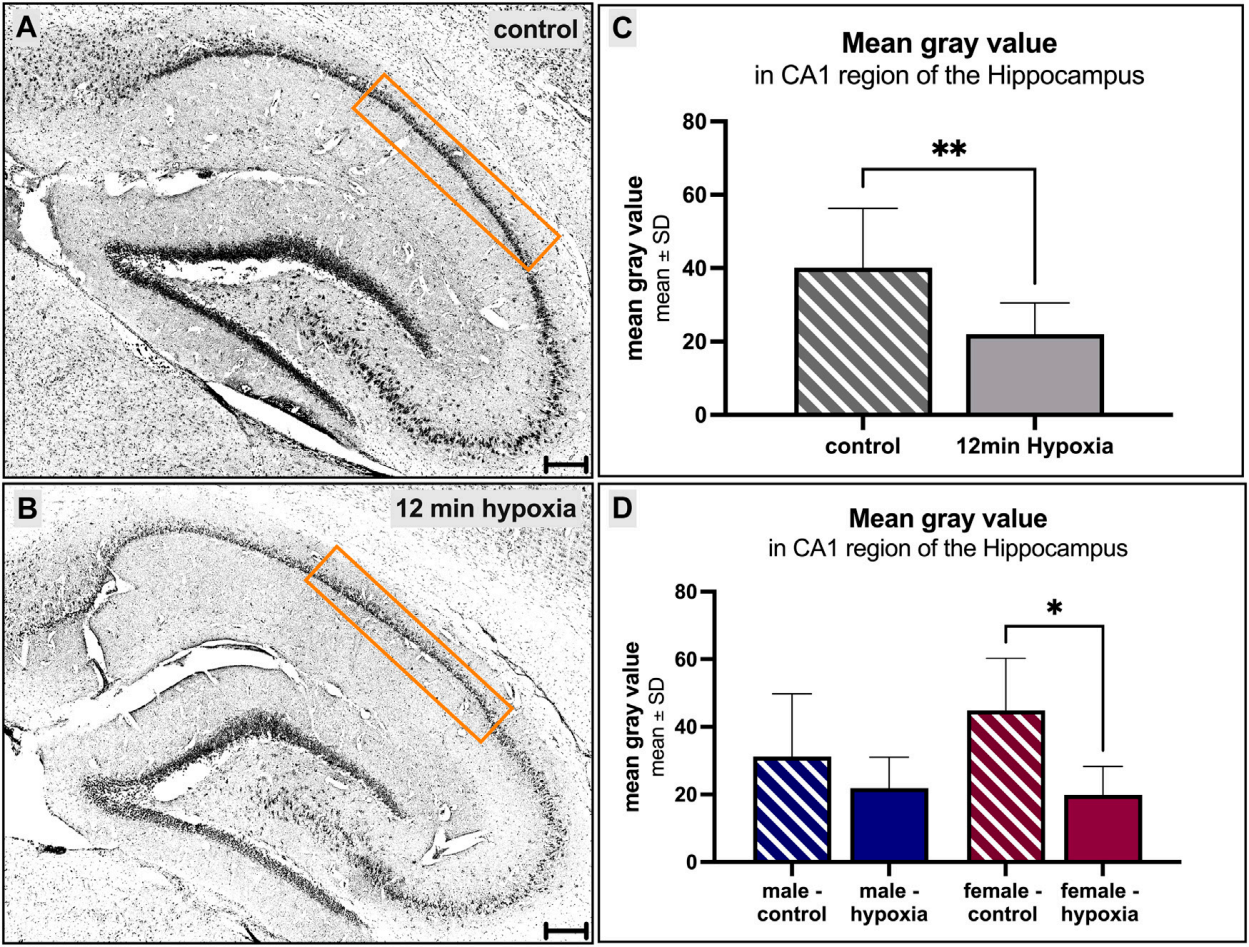
Morphological alterations in the hippocampus after cerebral hypoxia. **(A, B)** showing cresyl violet-stainings, converted to grayscale. A prominent decrease of the number of cells within the CA1 region of the hippocampus under normal [**(A, C, D)** striped] and hypoxic [**(B–D)** filled] conditions is observed. Differences between the sexes were not seen **(D)**. Scale bar in **(A, B)** 200 µm.

The hippocampus has previously been investigated for its susceptibility after hypoxic events, and the CA1 region seemed to be particularly vulnerable. Thus, we focused on this region and analyzed the mean gray value, which may resembles the cell number or cytotoxic brain edema influencing tissue volume without necessarily causing cell loss, leading to changes in the gray value without affecting the total cell number. We found a significant (*p* = 0.01, Figure 3C) decrease in the mean gray value in animals suffering cerebral hypoxia (Figure 3B) compared to controls (Figure 3A). When the animals were subdivided according to their sex, this observation was also found for the males, with a mean gray value of 31.22 ± 18.56 in controls compared to 21.91 ± 9.14 under hypoxic conditions (Figure 3D) but only for the female rats this finding was significant (*p* = 0.015).

#### 4.2.2 Changes in the cerebellum

The prominent Purkinje cells are specific to the cerebellum and can be found in large numbers and regular intervals. They are remarkable due to their arrangement in line, their massive flat dendritic trees, and their overall size.

**FIGURE 4:**
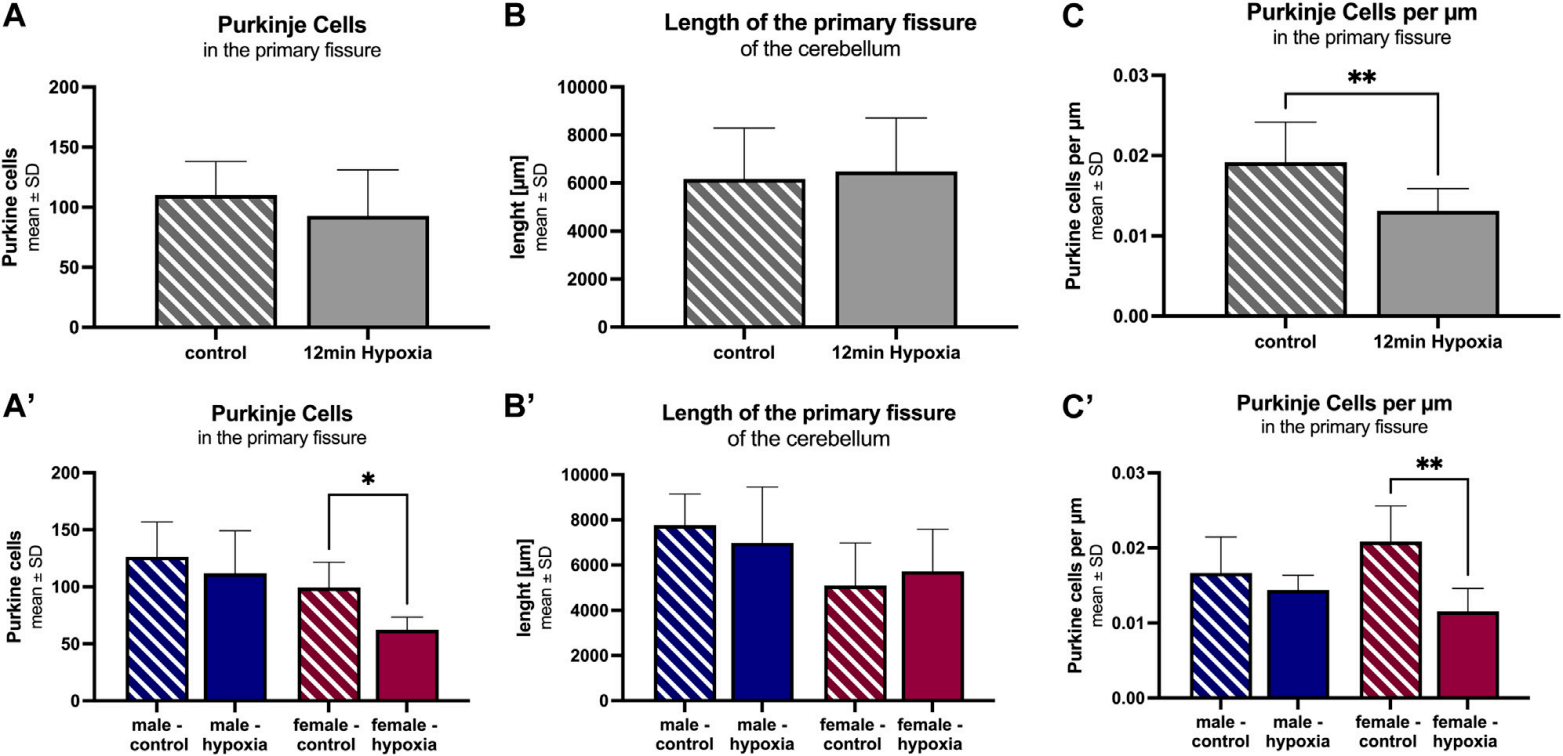
Morphological alterations in the cerebellum after cerebral hypoxia. The number of Purkinje cells within the primary fissure in animals undergoing anesthesia only (striped) is significantly higher than in those animals suffering global cerebral hypoxia (filled). The total cell number **(A)**, the length of the primary fissure **(B)**, and Purkinje cells per µm along the primary fissure are given **(C)**. The corresponding figures **(A–C)** demonstrate a more detailed analysis for the parameter “sex”. All values are presented as mean ± standard deviation. Significance was calculated using the unpaired *t*-test. A *p*-value <0.05 was considered significant (*; *p* < 0.001 **).

Under both control and hypoxic conditions, numerous Purkinje cells were seen along the primary fissure. However, the total number of Purkinje cells tends to be lower in hypoxic animals (92 ± 38 cells) compared to controls (110 ± 28 cells) (Figure 4A). After adjusting the number of cells to the measured length of the primary fissure (6.2 ± 2.1 mm and 6.4 ± 2.2 mm, respectively; Figure 4B), it was clearly seen that the number of Purkinje cells per µm decreased significantly in hypoxic animals compared to controls (12 min hypoxemia: 13 cells/mm vs control: 19 cells/mm; *p* = 0.007) (Figure 4C).

A differentiation according to sex showed a lower total number of Purkinje cells along the primary fissure, as well as a shorter length of the primary fissure in females (99 ± 22 cells and 5.1 mm ± 1.9 mm, respectively) compared to males (126 ± 31 cells and 7.8 mm ± 1.4 mm, respectively), but without statistical significance. However, a detailed analysis revealed a pronounced Purkinje cell decrease in hypoxic females compared to control females. This applied both, for the total number of Purkinje cells in the primary fissure (Figure 4A; *p* = 0.016), and for the number of Purkinje cells adjusted to the length of the fissure (Figures 4B, C; *p* = 0.003), indicating that Purkinje cells in female rats are significantly affected by hypoxia.

**FIGURE 5:**
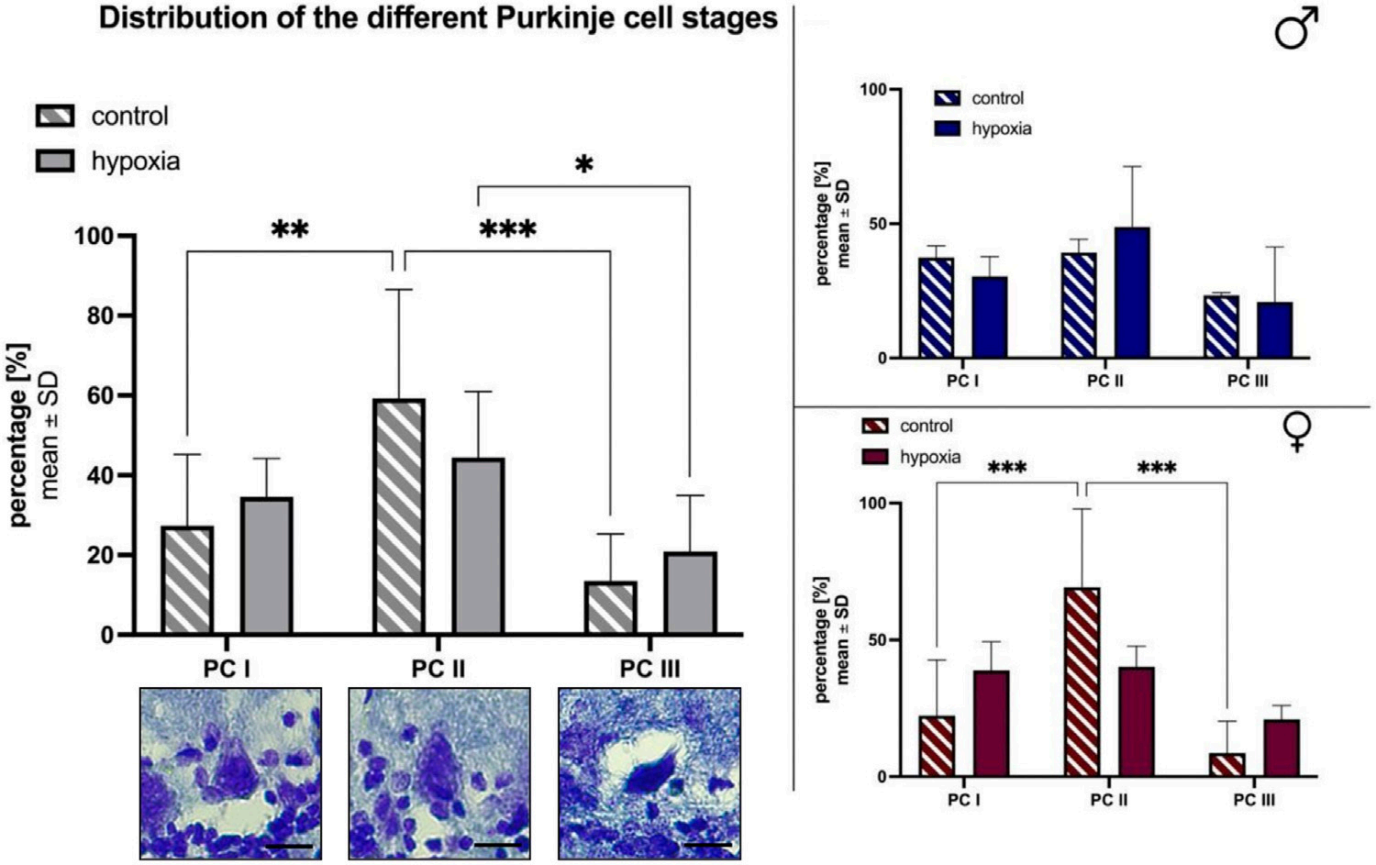
Morphological alterations in Purkinje cells after cerebral hypoxia. The percentual total cell distribution. The photographs depict the three distinct states of cell degeneration in hypoxic rats. The scale bar has been standardized to 20 µm for reference **(A)**. Also a subdivision between male **(B)** and female **(C)** are shown. All values are presented as mean ± standard deviation. Significances were calculated using two-way ANOVA with Sidak’s multiple comparison test. A *p*-value <0.05 was considered significant (*; *p* < 0.001 **). PC = Purkinje cell bodies.

Furthermore, we divided the Purkinje cell bodies found on the serial sections of the primary fissure, into morphologically different stages as previously described by Hausmann et al. (2007). In brief, cells were differentiated between “normal” appearing Purkinje cell bodies (PC I), Purkinje cells mostly lacking nuclear staining (PC II), and highly granulated, dark stained ones (PC III; Figure 5A). Less than half of the Purkinje cells in control (27.37% ± 17.78) and hypoxic (34.59% ± 9.64) animals had a normal (PC I) appearance. Overall, animals of the control group had significantly less PC III cells compared to PC II (*p* < 0.001) and PC I (*p* = 0.002), whereas in hypoxic animals comparatively less PC I and PC II Purkinje cell bodies were found. Thus, the proportion of PC III cells was tendentially higher in hypoxic animals than in controls (hypoxia: 20.91% ± 14.03; control: 13.52% ± 11.76). Again, these effects were more prominent in females (Figure 5C) compared to males (Figure 5B).

Additionally, the perimeter, the maximal caliper and the shape of the Purkinje cell bodies were determined (data not shown). The perimeter and the maximal caliper of PC III cells were significantly lower compared to PC I and PC II cells in both groups, and without significant differences between PC I and PC II cells. The shape of the Purkinje cell bodies (elongated or round) was comparable throughout all cell stages and both groups.

### 4.3 Hypoxia markers in blood 24 h after hypoxemia

Since blood has a high diagnostic value, it was collected 24 h after hypoxemia and screened for markers of hypoxia and other possible downstream effects as inflammation and migration after cell damage.

**FIGURE 6:**
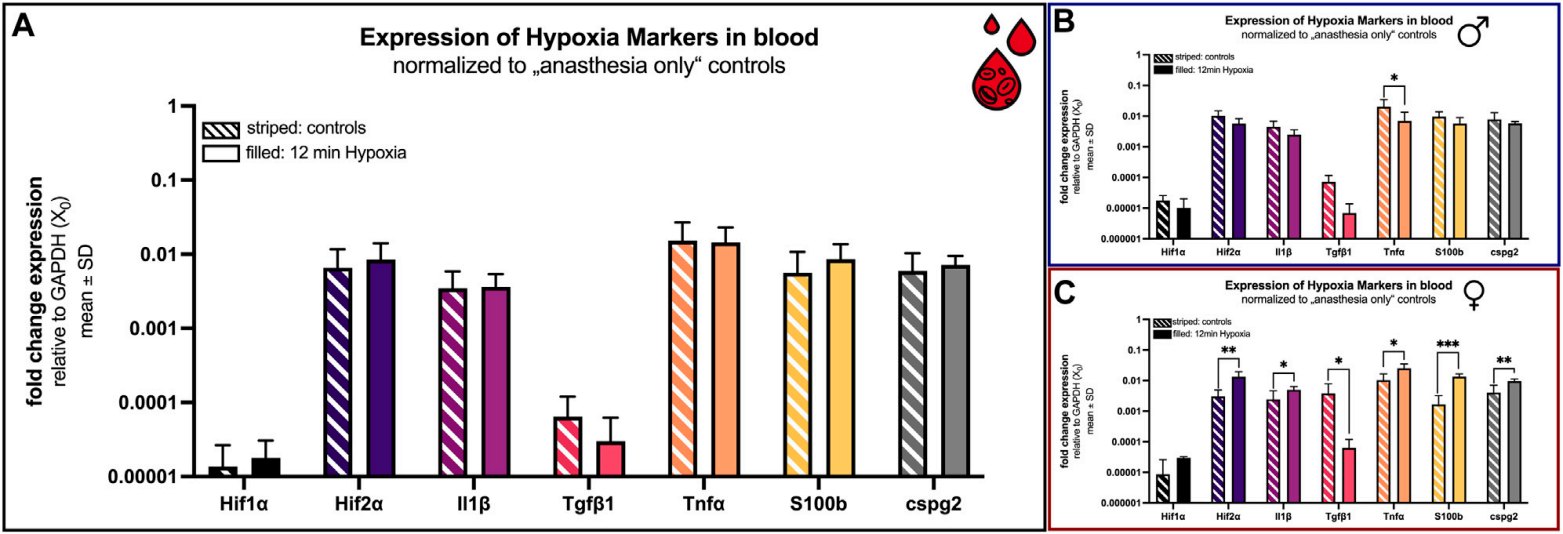
mRNA expression of hypoxia blood markers. Comparison 24 h after hypoxemia (filled) and in animals with anesthesia only (striped). **(A)** all animals; **(B)** males only; **(C)** females only. Differences were calculated using the unpaired *t*-test. All values are presented as mean ± standard deviation (log scale). A *p*-value <0.05 was considered statistically significant (*; *p* < 0.001 **; *p* < 0.0001 ***).

First, concentrations of both isoforms of the hypoxia-inducible transcription factor (*Hif*) *Hif 1-alpha* (*Hif1α*) and *Hif 2-alpha* (*Hif2α*) were analyzed. Concentrations were 0.14 × 10^−4^ ± 0.13 × 10^−4^ (*Hif1α*) and 65.8 × 10^−4^ ± 50.2 × 10^−4^ (*Hif2α*) in the control group and did not differ significantly after 12 min of hypoxemia (Figure 6A). However, a sex-specific subgroup analysis revealed that the *Hif1α* and *Hif2α* concentration in hypoxic females, was higher than in control females, while the reversed effect was seen in males (Figure 6B, C). The difference between hypoxic females (13.5 × 10^−3^ ± 6.04 × 10^−3^) and control females (3.04 × 10^−3^ ± 1.88 × 10^−3^) was statistically significant (*p* = 0.0037) regarding *Hif2α*.

Additionally, concentrations of proinflammatory cytokines like *interleukin-1 beta* (*Il1β*), *transforming growth factor beta 1* (*Tgfβ1*), *tumor necrosis factor alpha* (*Tnfα*), *chondroitin sulfate proteoglycan 2* (*Cspg2*) and the *S100 calcium-binding protein B* (*S100b*) were comparable considering all animals (Figure 6A). If, however, the sex is included, a significant difference is evident for all examined markers in the females (Figure 6C).

The concentrations of *Il1β*, *S100b* and *cspg2* were significant elevated (*p* = 0.039, *p* < 0.0001 and *p* = 0.009) in female rats that underwent hypoxia (4.98 × 10^−3^ ± 1.42 × 10^−3^; 13.6 × 10^−3^ ± 2.91 × 10^−3^ and 9.69 × 10^−3^ ± 1.64 × 10^−3^) compared to control females (2.44 × 10^−3^ ± 2.11 × 10^−3^; 1.69 × 10^−3^ ± 1.53 × 10^−3^ and 4.09 × 10^−3^ ± 2.96 × 10^−3^; Figure 6C).

The *transforming growth factor beta type 1* (*Tgfβ1*) was overall low in both groups (control: 6.43 × 10^−5^ ± 5.56 × 10^−5^; hypoxia: 2.99 × 10^−5^ ± 3.24 × 10^−5^) (Figure 6A). However, it is the sole marker showing the same tendency (downregulation after hypoxia) in both sexes. For females again, this observation was significant (*p* = 0.045). *Tgfβ1* was lower in hypoxic animals (6.29 × 10^−5^ ± 5.57 × 10^−5^) compared to controls (380.5 × 10^−5^ ± 405.8 × 10^−5^; Figure 6C).

Also unique was the expression of *Tnfα*. While globally no significant difference in expression was observed (control: 15.19 × 10^−3^ ± 11.74 × 10^−3^; hypoxia: 14.46 × 10^−3^ ± 8.55 × 10^−3^), a significant change was observed in both cases when considering the sexes individually. Thereby, *Tnfα* is significantly reduced in male rats after hypoxia (*p* = 0.049; control: 20.08 × 10^−3^ ± 14.37 × 10^−3^; hypoxia: 6.86 × 10^−3^ ± 6.39 × 10^−3^). Conversely, the expression is significantly increased in females after hypoxia (*p* = 0.011; control: 10.30 × 10^−3^ ± 6.27 × 10^−3^; hypoxia: 25.12 × 10^−3^ ± 9.83 × 10^−3^; Figure 6).

The expression of the clinical marker neuron-specific enolase (NSE) shows an incomparable increase (control: 0.0 ± 0.0; hypoxia: 4.39 × 10^−5^ ± 5.17 × 10^−5^). NSE could only be detected after hypoxia, not in control rats. Thus, NSE is significantly (*p* = 0.0039) increased in both sexes.

### 4.4 Hypoxia markers in the hippocampus and cerebellum 24 h after hypoxemia

#### 4.4.1 Effects in the hippocampus

**FIGURE 7:**
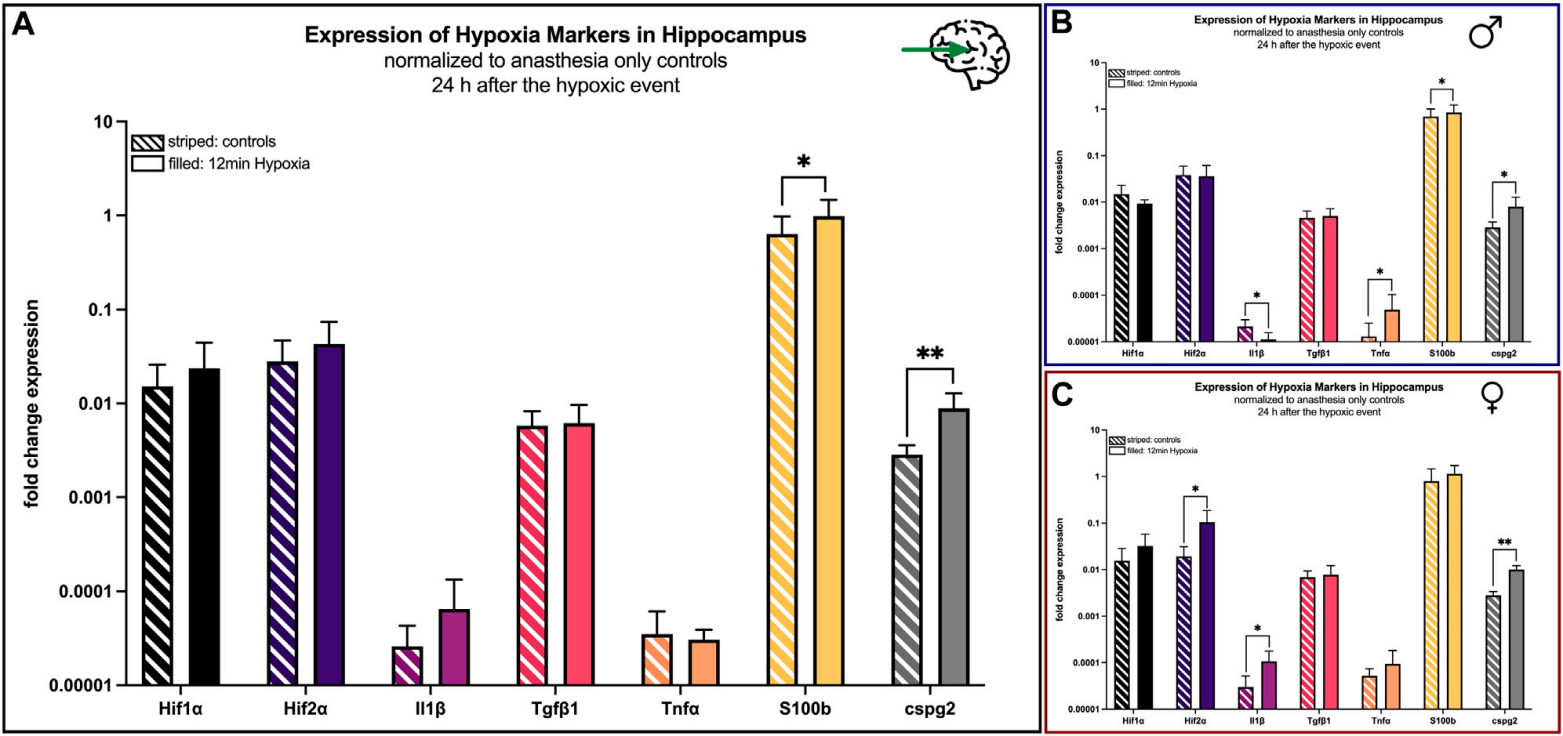
mRNA expression of hypoxia markers in the hippocampus 24 h after hypoxemia (filled) and in animals with anesthesia only (striped). **(A)** all animals; **(B)** males only; **(C)** females only. Differences were calculated using the unpaired *t*-test. All values are presented as mean ± standard deviation (log scale). A *p*-value <0.05 was considered significant (*; *p* < 0.001 **).

The above-mentioned hypoxia markers were also investigated in the hippocampus and the cerebellum 24 h after hypoxemia. In the hippocampus all markers were elevated in the hypoxia group compared to controls. Differences were statistically significant for *S100b* (*p* = 0.048), *cspg2* (*p* < 0.001) and NSE (*p* = 0.029) (Figure 7A).

Looking at males only, *Il1β* was significant (*p* = 0.03) reduced after the hypoxic event, whereas the markers *Tnfα* (*p* = 0.041) and *cspg2* (*p* = 0.041) revealed higher concentrations after the hypoxic event (Figure 7B).

In contrast, all hypoxia markers in the hippocampus were upregulated in female hypoxic animals compared to controls with statistically significant differences for *Hif2α* (*p* = 0.02), *Il1β* (*p* = 0.018) and *cspg2* (*p* = 0.002) (Figure 7C).

#### 4.4.2 Effects in the cerebellum

**FIGURE 8:**
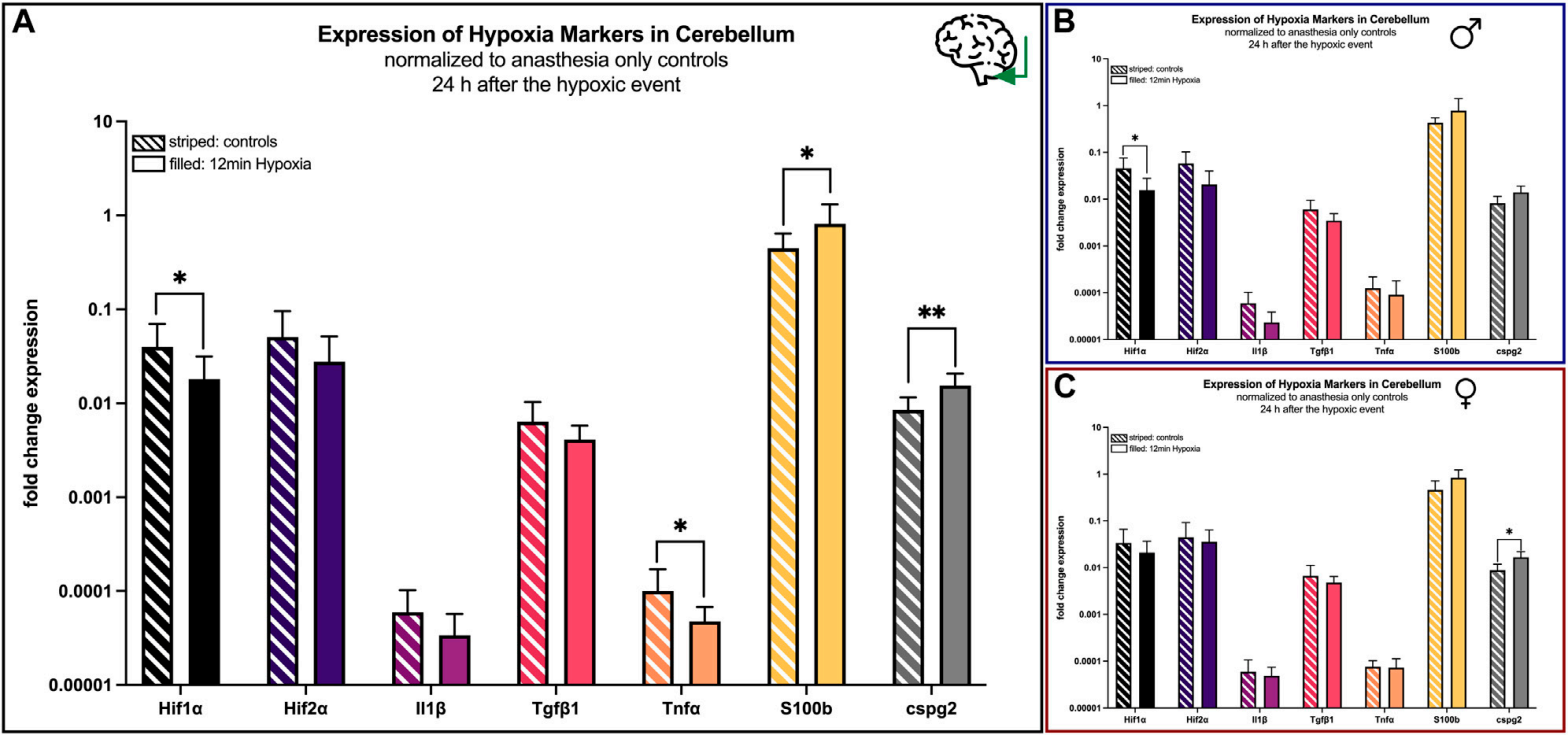
mRNA expression of hypoxia markers in the cerebellum 24 h after hypoxemia (filled) and in animals with anesthesia only (striped). **(A)** all animals; **(B)** males only; **(C)** females only. Differences were calculated using the unpaired *t*-test. All values are presented as mean ± standard deviation (log scale). A *p*-value <0.05 was considered significant (*; *p* < 0.001 **).

In the cerebellum the expression of the markers *Hif1α* (*p* = 0.041), *Tnfα* (*p* = 0.045), S*100b* (*p* = 0.029), *cspg2* (*p* = 0.001) increased significantly in animals undergoing hypoxemia compared to controls, whereas the regulation of the other markers remained heterogeneous (Figure 8A).

However, as demonstrated above, a sex-specific analysis demonstrated nearly no significant changes in males, except from *Hif1α* (*p* = 0.047) being lesser expressed after 12 min of hypoxia (Figure 8B), whereas differences in females were only significant for and *cspg2* (*p* = 0.017) (Figure 8C).

## 5 Discussion

Just like other organs, the brain is dependent on a continuous supply of oxygen and nutrients to ensure normal function, however, its cells are particularly vulnerable to any oxygen deficit. Hypoxemia, which leads to cerebral hypoxia, can affect individuals of all ages and may - regardless of its etiology - cause fulminant brain damage, often accompanied by severe lifelong disability or death.

### 5.1 Global cerebral hypoxia induced by rocuronium

In this manuscript we presented an innovative minimally-invasive rat model, which mimics the pathophysiology of global cerebral hypoxia, as it creates hypoxemia and consecutive cerebral hypoxia, while the blood flow is still preserved. We demonstrated that 1) hypoxia can be gained by using the muscle relaxing agent rocuronium (severe respiratory insufficiency), 2) hypoxia can be maintained for at least 12 min, and 3) hypoxia can be reserved within 1–3 min by use of sugammadex. The functionality was morphologically proven by a significant reduction of neurons in the hippocampus and a decrease of Purkinje cells in the cerebellum, as well as biologically by significant changes in hypoxia markers in blood and brain tissues 24 h after hypoxemia.

According to the oxygen hemoglobin dissociation curve an oxygen saturation of 50% corresponds to an arterial oxygen partial pressure of approximately 30 mmHg, which leads to bradycardia, hypotension, and is generally considered to be lethal (Sakamoto et al., 1991). We chose a 12-min interval of hypoxemia with an SaO2 of about 60%, as this is on the one hand long enough to expect an irreversible neurological damage, but on the other hand not too long to safely survive the next 24 h (Schneider, 2009). Moreover, rocuronium’s duration of effectiveness must be considered, which is short (a few minutes up to 1 hour). A repetitive rocuronium injection to maintain respiratory failure after spontaneous recovery should be avoided, as its effect can hardly be titrated. Thus, we focused on 12 min in this pilot study, although shorter or longer time intervals up to approximately 30 min are technically possible.

### 5.2 Morphological aspects

The CA1 region of the hippocampus is known to be vulnerable to oxygen deficiency and was chosen, because cell degeneration was previously observed in humans as well as in animals suffering cerebral hypoxia (Schmidt-Kastner and Freund, 1991; Bartsch et al., 2015). Indeed, our findings were comparable to those observations in humans and in rats, although the cell degeneration in these studies was most pronounced between the third and seventh day after the hypoxic event, indicating that cell decrease in our model could have been worsen at a later point in time (Schmidt-Kastner and Freund, 1991; Bartsch et al., 2015).

The above demonstrated morphological alterations and the extent of Purkinje cell death in our model also corresponds to observations made in humans and hypoxia rat models by others (Barenberg et al., 2001). Biran and colleagues, for example, exposed rats against 5.5% oxygen for 2.5 h. Animals demonstrated severe pathological findings, including a decreased thickness of the granular and molecular layers, diffuse white matter damage, and a decreased number of interneurons, pointing out the cerebellar vulnerability to hypoxia (Brasko et al., 1995). Again, overall results indicate that morphological irregularities were most pronounced about 1 week after hypoxia, meaning that the neuronal degeneration we observed 24 h after hypoxia would probably still go on (Brasko et al., 1995) (Bartschat, 2012; Biran, 2011; Taniguchi, 1994). Also, the distribution of Purkinje cells did not significantly change when comparing the control and hypoxia groups. Sato et al. suggested that this lack of change may be due to delayed neuron death, where visually recovering cells degenerate after about 4 days. Since our animals were terminated after 24 h, further investigation with animals terminated a few days later would be of interest in the future (Buisson et al., 2003).

It is of note that this model is not specific to cerebral hypoxia, as all other organs and tissues are affected by hypoxemia as well. However, the greatest damage is expected in the brain, as its cells are most sensitive to a lack of oxygen.

### 5.3 Hypoxia blood markers

Hypoxia markers were primarily used to validate the model; however, blood markers are also useful from a clinical perspective, as blood analysis is a highly suitable method due to its ease, speed, progress control and minimal invasiveness for patients. Therefore, the identification of hypoxia markers in blood would hold significant diagnostic value and possibly predict outcome in humans.

Hypoxia leads to an activation of cascades including inflammation, reoxygenation, and production of reactive oxygen species (Peng et al., 2013). Thus, not all markers were hypoxia-specific; we also included inflammatory markers to demonstrate the well-established connection between inflammation and hypoxia in various biological processes (Malkov, 2021; Thornton 2000). It is known that the activation of cascades happens sequentially, meaning that corresponding markers cannot be determined at one ideal point in time. In this first approach we examined blood and brains 24 h after hypoxemia, because studies demonstrated that the tested markers could be detectable at that time.

*Hif* proteins are transcription factors and regulate the adaptation to low oxygen conditions in humans and animals by expression of various hypoxia-dependent genes, which are, among others, involved in energy metabolism, angiogenesis, and cell survival (Zhang and Yang, 2020). In our experiments *Hif2α* had higher mRNA expression levels compared to *Hif1α*. The expression levels did not differ between control rats compared to rats that underwent 12 min of global cerebral hypoxia, except looking at only female rats, where significant elevated mRNA levels of *Hif2α* could be detected in the hypoxic animals. Although both factors have very large structural homologies, Park et al., 2003 showed that they can be regulated differently and are not redundant (Missler et al., 1997). Additionally it is known that *Hif2α* is more stable than *Hif1α* (Pan and Kastin 2007).

The *transforming growth factor Tgfβ1* is known to be involved in neuroprotection after cerebral hypoxia and ischemia, and has been previously discussed in blood (Yan et al., 2022) and the context of multiple sclerosis (Wight et al., 2020), ischemic brain injury (Asplund et al., 2010; Dhandapani, 2003), stroke (Paulus et al., 1996), and cerebrovascular diseases (Hernández et al., 2021). Although an increase of this marker after hypoxia is assumed, a high concentration of *Tgfβ1* does not necessarily correspond to a beneficial outcome, as some studies even demonstrated a higher degree of neuronal cell death (Wiener et al., 1996). Moreover, a cut off value has not yet been defined. We observed a significantly lower *Tgfβ1* concentration in hypoxic female rats compared to controls, which we interpreted as involvement in neuropathology.

The astroglial protein *S100b* is a cytosolic calcium-binding protein It is mainly expressed in astrocytes but also in neurons, from which it is secreted after hypoxia (Bloomfield, 2007, Ellis, 2007; Salceda and Caro, 1997; Winter, 2014). Studies demonstrated neurotrophic and neuroprotective effects of *S100b*, however, increased concentrations are also associated with tumor progress and the progression of neurodegenerative diseases (Boche et al., 2013; Michetti, 2019). We detected significantly elevated mRNA blood levels for female rats only. However, it should be noted that peak concentrations are to be expected on day 2.5 ± 1.3 after hypoxia.

NSE is a widely used clinical biomarker for post resuscitation care and after other hypoxemic events. Unlike S100b, it is located in neuronal bodies. Blood levels of NSE rise up to 24–48 h after a hypoxemic event. Due to a half-life of 24–30 h, NSE can be detected in blood 4–5 days after a hypoxic brain injury depending on the severity (Sandroni et al., 2023). Increased NSE levels can also be caused by malignant proliferation and neurodegenerative diseases (Isgrò et al., 2015). We found a significant increase in NSE in both sexes.

### 5.4 Hypoxia markers in the hippocampus and the cerebellum

In addition to blood examination, we also evaluated the markers described above in the hippocampal and cerebellar regions of the brain that are known to be susceptible to hypoxia.

Wiener and colleagues demonstrated high concentrations of *Hif* mRNA in tissue culture cells of humans and rats, as well as *in vivo* in rats and mice after exposure to normobaric hypoxia with 7% oxygen for 30–120 min (Berti et al., 2002). Increased *HIF* concentrations were also seen in various other hypoxia studies, however, concentrations were almost always determined directly after the hypoxic event or after a long chronic exposure to low oxygen levels as described in the endogenous chronic anemia model of hypoxia (Ajmone-Cat et al., 2013). In addition, it is known that *Hif1α* reacts very fast and the *Hif1α* expression is cell specific (Krupinski, 1996). Moreover, *Hif1α* was also not detectable in human *postmortem* brain tissue during autopsy after proven lethal primary hypoxia, indicating that the chosen time point in our experiments was probably too late (Schneider, 2009; Guerrero and McCarty, 2017).

The mRNA concentrations of *S100b* and *cspg2* were increased in both, hippocampus and cerebellum, whereas the concentration of *IL1β* and *NSE* was increased in the hippocampus only.

As mentioned above, *S100b* is an interesting biomarker for hypoxia in the central nervous system, as its concentration rises hours before neuroimaging or neurological examinations show pathological results. Moreover, normal *S100b* concentrations suggest even the absence of significant neuronal injury (Luo, 2022). In our model *S100b* concentrations were elevated in the hippocampus and in the cerebellum, which is consistent with findings from hypoxia cell models (Clayton and Collins, 2014) and from humans being exposed to hypobaric hypoxia (Klein et al., 2015).

The proteoglycan *cspg2* was chosen, because this extracellular matrix molecule is associated with inflammation, brain tumors, and has been proven to be secreted by macrophages as a response to hypoxia (Brambrink et al., 1999; Crain et al., 2013; Tannenbaum et al., 2016). Its mRNA increase in different brain areas underlines the efficiency of our model.

The cytokine *IL1β* is released by microglia during early stages of hypoxia, which leads to inflammation or cell death (Bartsch et al., 2015). It was chosen, because its concentration is known to rise in hypobaric hypoxia models, which fits our results (Zhang and Yang, 2020).

The *neuron-specific enolase (NSE)* is an important enzyme in glucose metabolism, which can get into the blood after damage to neurons or the blood-brain barrier. Thus, it can be detected in blood as an ideal marker for brain injury (Hernández et al., 2013). In addition to the significant increase in NSE in the blood as shown above, we were able to detect the primary brain injury in the hippocampus.

### 5.5 Hypoxia markers and timing

*Tnfα* is a marker of inflammation and involved in the migration process of cells from oxygenated tissues to hypoxic-inflamed brain regions; its production is partly regulated by the concentration of *Hif1α* (Bartsch and Wulff 2015; Zhang and Yang 2020). However, although elevated *Tnfα* levels had been described in the context of hypobaric hypoxia in mice (Pan et al., 2020), it is rather discussed in the context of cerebral ischemia (not hypoxia) (Hausmann et al., 2007). Thus, the observed lack of *Tnfα* upregulation in our study is not surprising, as we had no increased *Hif1α* levels and no cerebral ischemia. In addition, *Tnfα* concentrations are highest about 6 h after the hypoxic event and the concentration decreases rapidly after 24 h (Hausmann et al., 2007; Molina et al., 2017).

Lastly, no upregulation was seen for tissue *Tgfβ1*. This result is comparable to observations made by Molina and colleagues, who stated that *Tgfβ1* dropped in the hypoxia rat model shortly after the event compared to controls, while no changes at the protein level were detected (Molina et al., 2017).

### 5.6 Sex differences

For various reasons male rats are preferably used in experiments and transferability of results to female animals is often simply assumed, although studies have proven sex-specific differences (Clayton and Collins, 2014; Klein et al., 2015; Tannenbaum et al., 2016). Crain and colleagues, for example, showed that the expression of markers such as *Tnfα*, *IL1β*, and others depends on the sex, which is in line with our results for *Tgfβ1*, *S100b*, and *cspg2* (Torres-Cuevas et al., 2019). Additionally, we found morphological differences, such as a lower total number of Purkinje cells along the primary fissure in females. This may be explained by their generally smaller body size but needs to be considered. All in all, our findings emphasize the need for gender-based research. Also, a deeper investigation including the female cycle could be of interest in further experiments. In any case, it was demonstrated that the presented model is suitable for all sexes.

### 5.7 Potentials and limitations

The main advantage of this endogenous model is that it allows one to focus on the effect of hypoxia in the brain in a pathophysiological correct manner, while duration and severity of hypoxia is well-controllable without direct cerebral intervention. Thus, it is less time-consuming, invasive, and stressful for the animals, which, in addition, recover quicker than (surgically) instrumented rats. Moreover, the technique is easy to learn, the experimental setup is simple, the costs are comparably low, and no interaction or other pharmacological long-term effect of rocuronium must be feared, as it is encapsulated by sugammadex and excreted. However, controlling the depth of anesthesia is challenging. If anesthesia is too deep, animals suffer complete respiratory arrest after rocuronium administration, and the rats die immediately. If anesthesia is not deep enough, a severe tachycardia develops after the animals obtain rocuronium; they die due to heart failure before the endpoint is reached. Furthermore, the speed of rocuronium injection should be adapted to the rat’s individual response. It should be noted that a rocuronium dosage of 40 mg per kilogram for relaxing the diaphragm is high, especially since others reported a dose of 4 mg per kilogram to relax the femoral muscles in rats (Torres-Cuevas et al., 2019). Thus, animals must be monitored very well even after antagonization with sugammadex to ensure that they do not suffer a residual neuromuscular blockade and die within the first hour after the experiment. However, all animals, which reached the oxygen baseline levels after sugammadex administration survived until termination after 24 h.

Another benefit is that neither a typical reperfusion injury occurs (no occlusion of blood vessels), nor that overlying side-effects from the intervention have to be feared (Khatun et al., 2021). However, the reoxygenation after application of sugammadex (physiologically through inhalation of room air with 21% oxygen and supported via oxygen facemask) may lead to oxidative stress with mitochondrial dysfunction, neuroinflammation, or even cell death (Torres-Cuevas et al., 2019). Nevertheless, it should be noted that our rats were already 16 weeks old, and thus not newborn.

The time point of termination is another aspect, which should be discussed. This study was a pilot study to test and validate the functioning of the model. In addition to the chosen time point, termination of the animals at three, five, or 7 days was considered to potentially yield more pronounced results, allowing for the examination of longer-term effects. However, the termination of rats at four or 12 hours could also provide valuable insights, as these early time points capture acute pathophysiological responses. The inclusion of these time points would enable a comprehensive evaluation of both acute and longer-term reactions in further studies. Due to the requirements of our animal experimentation licence, the inclusion of further time points of interest in this pilot study was limited. Therefore, the addition of complementary time points will need to be considered depending on future experiments. In addition, the effects of hypoxemia on other organs could easily be evaluated as well.

The examination on mRNA level allows a relatively broad range of hypoxia markers and their mRNA differences to be analysed for validation of the model. Although a change in the mRNA level does not necessarily correspond to a change in protein expression, it does indicate a change in protein biosynthesis. Therefore, in further experiments with a specific therapeutic approach the investigation of corresponding protein levels should be taken into account.

Nevertheless, we think that this model has the potential to analyze different aspects of global cerebral hypoxia, including morphological reactions after hypoxia and protein levels, sex- and age-specific differences, and - due to its minimally invasive character - maybe even the effect of comorbidities. However, this model was primarily intended for testing the potential of therapeutic and preventive drugs, which will be the focus in further experiments.

## Data Availability

The original contributions presented in the study are included in the article/Supplementary Material, further inquiries can be directed to the corresponding author.
