# Supplementary figures and images for: Innovative in vivo rat model for global cerebral hypoxia: a new approach to investigate therapeutic and preventive drugs
Source: Front Physiol. 2024 Feb 9;15:1293247. doi: 10.3389/fphys.2024.1293247 (PMC10885152; doi:10.3389/fphys.2024.1293247)

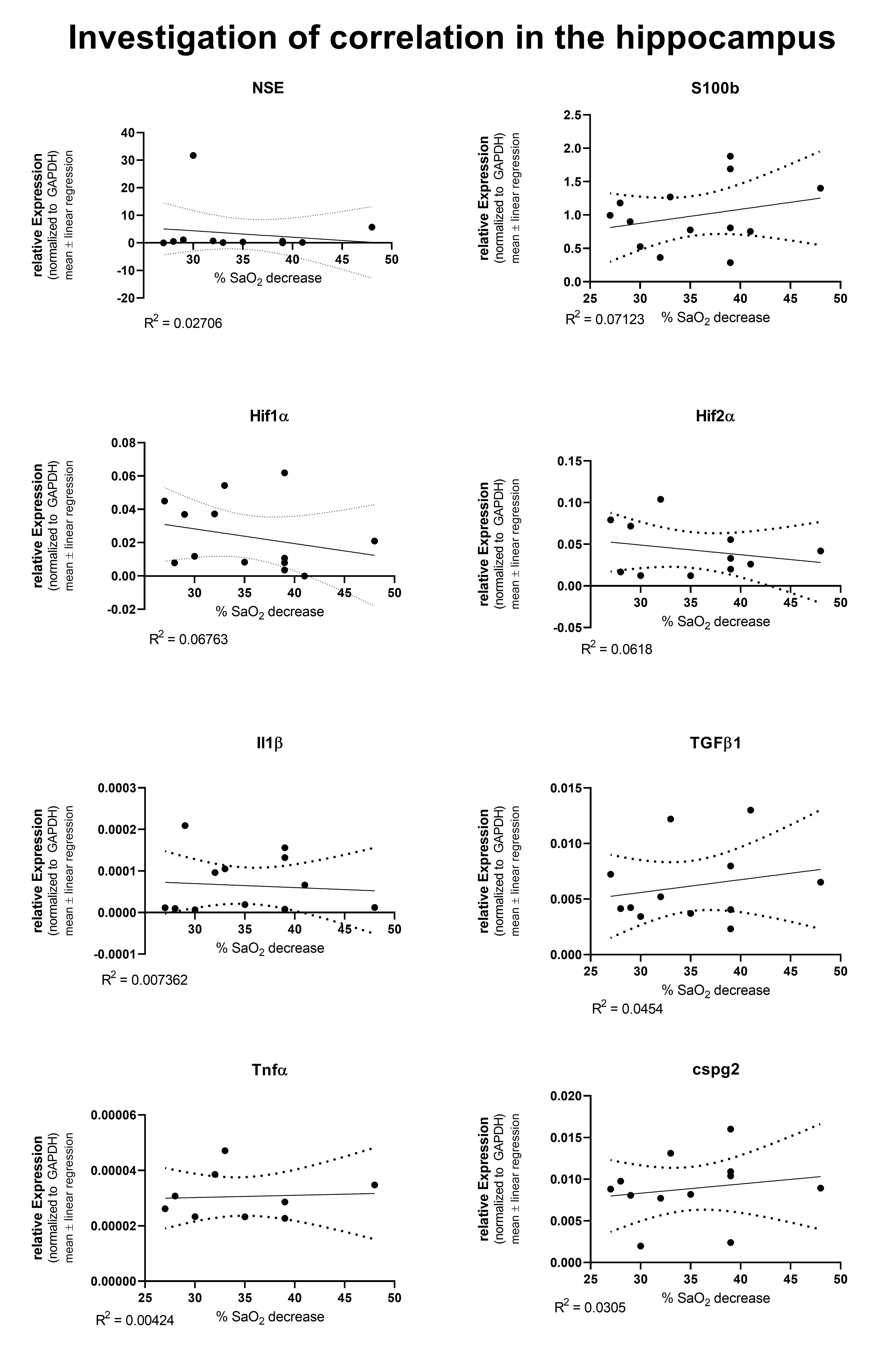

Supplement: Supplementary file 1 [file Image3.JPEG]

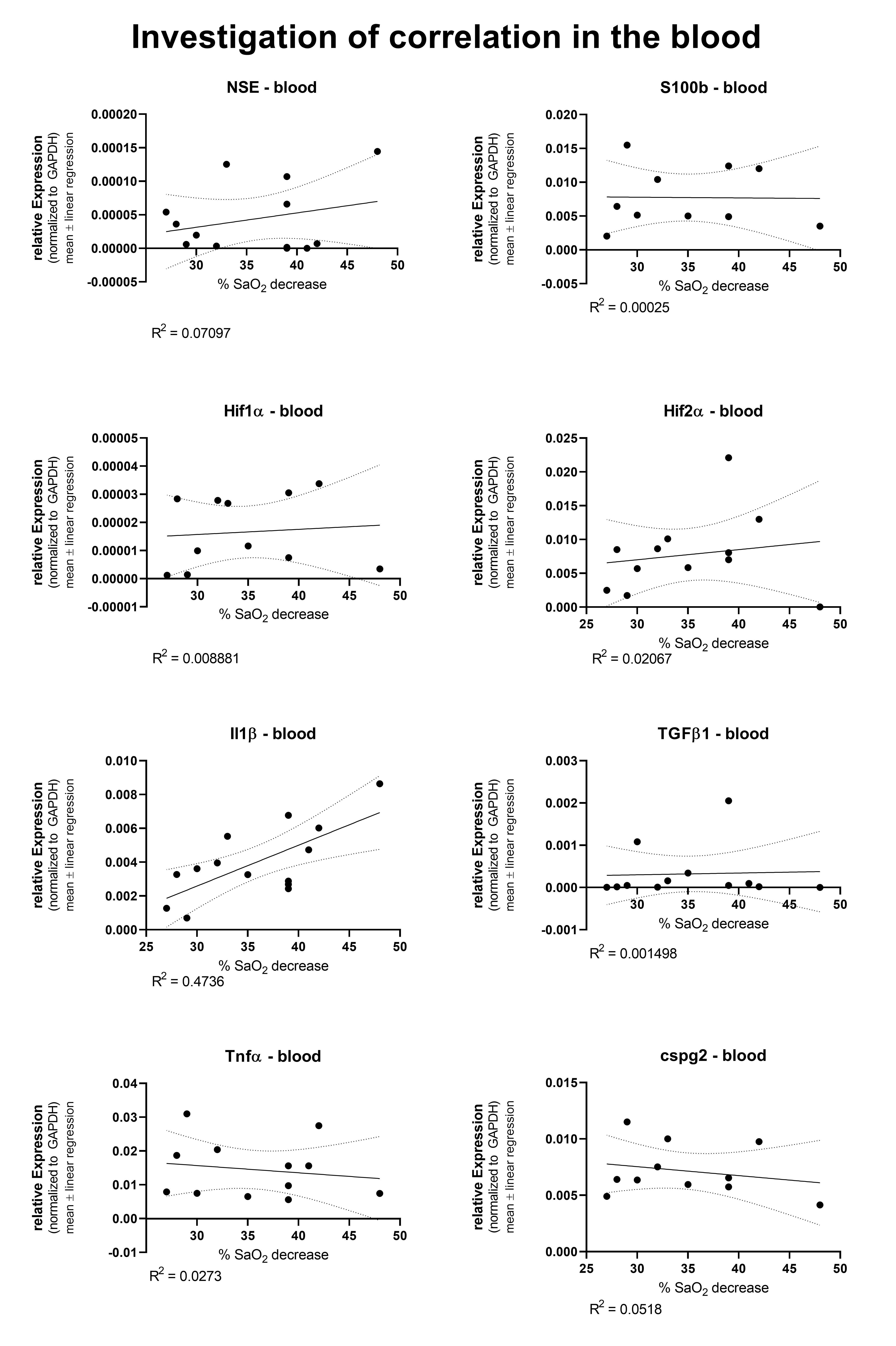

Supplement: Supplementary file 2 [file Image1.JPEG]

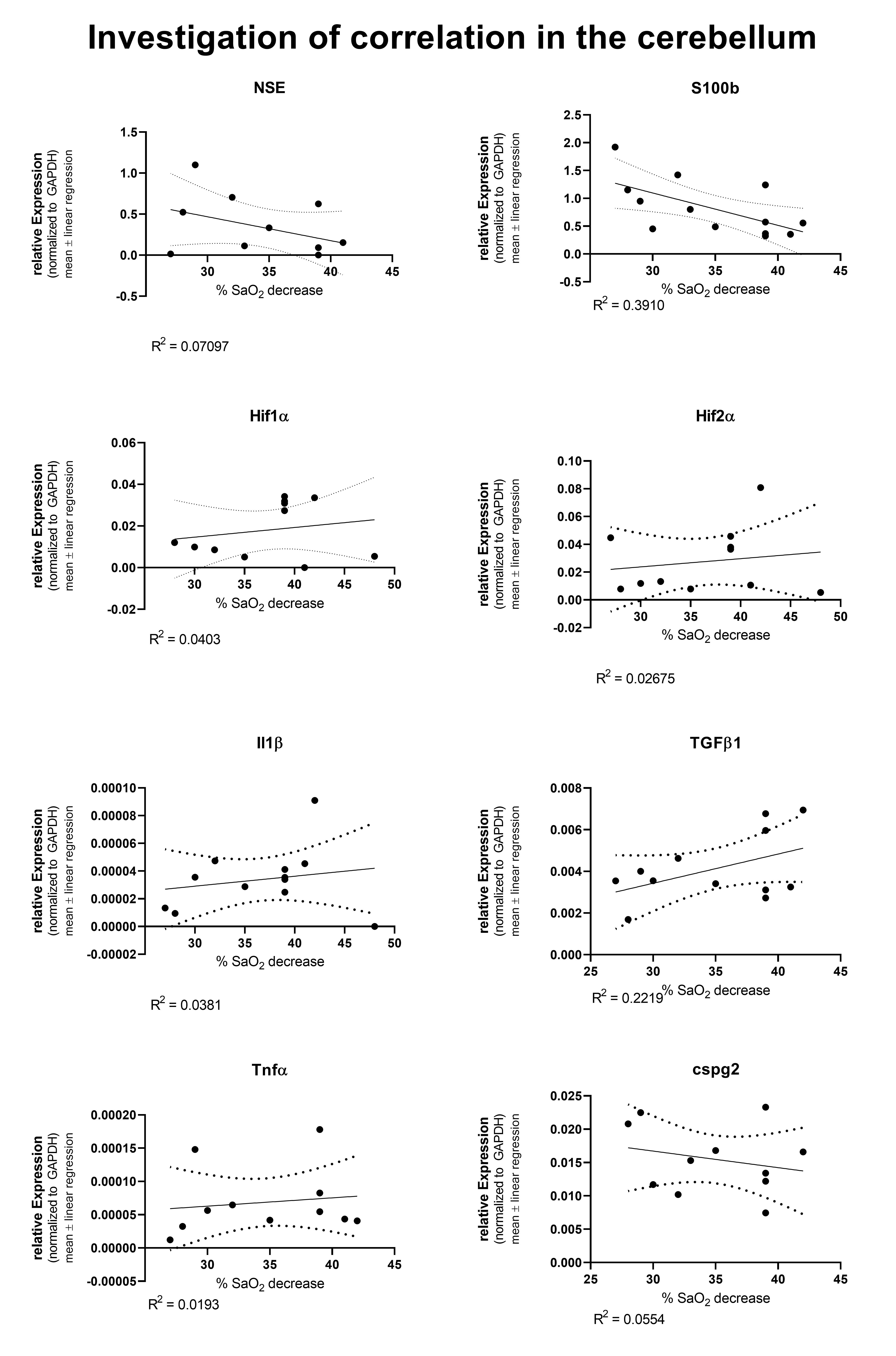

Supplement: Supplementary file 3 [file Image2.JPEG]
